# Young sociologists in the mirror: digital ethnographies of young people online

**DOI:** 10.3389/fsoc.2023.1197560

**Published:** 2023-08-24

**Authors:** Nadia Crescenzo

**Affiliations:** Department of Political and Social Studies, University of Salerno, Fisciano, Italy

**Keywords:** young people, netnografy, youth practices, online practices, qualitative method

## Abstract

The aim of the paper is to analyze how the qualitative method represents a tool capable of analyzing the youth experience in the digital context. The contribution takes as a starting point the results of a didactic exercise experimented in the course of “Sociology of youth cultures” by the students of the University of Salerno in which the qualitative method of netnography was applied to study some youth practices online. During the work carried out, the qualitative method represented a way to study the universe of young people, entering the “new digital habitats,” where young people create and reproduce relationships, identities and spaces for socializing. The results showed, first of all, how the method of netnography made it possible to analyze the contours and transformations of youth practices in digital spaces, and secondly it allowed to arouse “reflexivity” in young people, it allowed to activate a process in which young people have looked at themselves “in the mirror,” entering the folds of the virtual worlds of youth and are amazed at the way in which they represent themselves, becoming observers and observers of that same reality. The qualitative approach has therefore allowed the students to grasp the youth practices in which they are immersed in their daily life from another point of view and have had the opportunity to analyze them from the inside and develop a high degree of reflexivity that it helped to reach a different awareness of being young today and of being able to read the cultural dynamics of youth with a critical eye.

## 1. Introduction

The network today represents a pivotal space in people's lives, in particular those of the new generations. In their daily experiences, young people come into contact instantly - they relate with a simple click and communicate drawing on a multiform universe of images, emoticons, memes, posts, tags, hashtags (Chambers, 2013; Romeo, 2017; Pattaro and Setiffi, 2020). In so doing, they constantly produce and exchange meanings, representations and symbolic models (Bakardjieva, 2005; Riva and Scarcelli, 2016; Drusian et al., 2019; Amendola et al., 2020; Savonardo, 2020).

At the same time, new digital technologies involve the necessary redefinition of the objects of study of the social sciences, the tools and methods used to investigate, analyze and understand identities, institutions and social worlds, as well as the work of the sociologist.

Drawing on Lupton (2018), we can summarize the challenges posed by Digital Sociology along four key dimensions: the use of digital tools for the professional practice of the sociologist; the analysis of the uses of digital technologies and emerging meanings; the use of digital data in and for quantitative and qualitative research; the reflective and critical analysis of digital tools.

Departing from this perspective, this paper aims to analyze how the qualitative method represents a tool to explore the youth experience in the digital context. The essay unfolds along three specific levels of analysis: firstly, it analyzes the use of netnography for research on young people; secondly, it enhances the value of research with young people to study young people; finally, it illustrates the relationship between learning experience and research experience. The first level represents the context element of the paper while the second and third levels will be the central topics of the essay.

The starting point are the results of a didactic exercise carried out during the course of “Sociology of youth cultures” with students at the University of Salerno, in which the method of netnography was applied to study some youth practices online. The work involved 15 young students who participated in the course.

The hypothesis underlying the work was twofold: on the one hand, to offer students an opportunity to deal with the professional, methodological and theoretical challenges of digital sociology, so as to be able to adopt a reflective attitude on skills they are called to acquire; on the other, to invite them to sociologically observe the (digital) world in which they are immersed and within which they build their lives, their relationships and their maps of meaning. In other words, an attempt has been made to invite them to “look in the mirror”: that is, to put to the test the theoretical knowledge and methodological skills that they are called to acquire as students, assuming their own world as an object of analysis, that of young people whose online and offline lives intersect and overlap until they almost merge.

If, on the one hand, the path developed with the students showed the way in which being rooted in concrete places and spaces coexists with the “dematerialized” digital environment, on the other one, epitomizing an ethnography that becomes reflective, it highlighted the specificity of the gaze of young people (students) who observe their peers (that is, themselves).

By crossing this dual level, the students were able to experience what doing digital ethnography means and question how some youth cultural practices exist on a “liminal interval” between material and digital spaces that new media make available (Hine, 2000; Ruhleder, 2000; Anderson, 2004; Beneiro-Montagut, 2011).

## 2. The method of analysis

The methodology used in the work carried out with the students is an ethnographic approach applied in the digital environment.

The exponential pace at which the use of the network spread has not only changed the way people access local and global information, but has also influenced the way they create communities and build their own identity.

Online interactions find more and more space in everyday life experiences: it is a dimension that has now become so widespread and influential in our day-to-day that it can no longer considered virtual, but real in all respects (Boccia Artieri, 2011; Tirocchi, 2013).

Ethnography could not remain indifferent to this metamorphosis of social relations and various scholars begun to pay attention to the meaning that online spaces assume as a locus for life and interaction (Esposito, 2016; Masullo et al., 2020; Chiriatti, 2021).

Kozinets (2002, 2006, 2019) described this new field of application of ethnography with the neologism “netnography” referring to the study of online communities. He also distinguishes six characteristic elements of netnography: design; entrée (the entrance to the field); data collection; interpretation; procedures necessary to ensure ethical standards; research report. Digital research ethics is a major issue. Kozinets recommends following certain procedures: the researcher must make his presence clear, communicating his affiliations and intentions to community members; must ensure the anonymity of informants; must seek and take into account the feedback offered by community members; Finally, the researcher must maintain a precautionary position with respect to the use of the collected data, directly contacting the members of the community to ask for their permission whenever he wants to directly quote one of their texts or use a digital artifact potentially covered by copyright.

The advantages of this method consist in the less intrusiveness compared to an observation in the presence and this cancels the problem of reactivity; among other elements, the possibility of carrying out the observation in a time established exclusively by the researcher is also an advantage. The limitations of netnography derive instead from its narrower focus on online communities, the need for interpretive skills of the researcher and the lack of identifiers of informants present in the online context which leads to difficulties in generalizing the results to groups outside the community sample online (Anne-Marie et al., 2017; Costello et al., 2017).

The work carried out with students concerns the realization of four project works during which netnography was applied to the study of some youth cultural practices that take shape online.

The task followed a five-phase process:

- The first step consisted of a moment of reflective debate and discussion on the ways in which young people communicate online. This allowed to identify the many shapes that youth cultural practices take online and the channels that young people more frequently use for their dissemination;
- In the second phase the students chose a specific practice to be analyzed among those that emerged during the debate;
- The third phase consisted in making the netnography by referring to a shared “checklist” that was used by all groups;
- The fourth phase consisted in group analysis and presentation of results;
- The last phase coincided with individual reflection.

The tool used was inspired by Altheide (2000) approach to media analysis: the checklist developed by students was used to read the platforms chosen. The checklist was set up to detect information relating to: source of content, time references of the survey, phrases used, type of comments to posts or images, detailed description of images, profiles of users sharing content and users commenting.

The data analysis took place through a categorization process of the recurrent characteristics that emerged during the analysis.

## 3. The process of analysis

In this section of the paper, the following aspects will be analyzed: the topics addressed by the four groups of students, the channels used, the type of content they analyzed (texts, photos, and comments) and the analysis carried out (categorization of photos and texts also identifying the consistency between photos, text and comment).

As regards the nature of the online practices analyzed by the students, they autonomously decided which ones to focus on, even starting from those practices with which they were more familiar and which particularly attracted their attention in their daily lives as young users.

The first group investigated the phenomenon of “body shaming”, analyzing the Instagram profile of four female influencers. The students examined comments that contained mockery/insults on physical appearance via specific photos from their profiles. 32 photos and 60 comments were analyzed, the latter being categorized into 4 types of affronts: regarding physical appearance, personal/character, the use of cosmetic surgery, followers. In some cases support for comments came from a simple like or a response to the comment itself. The conclusions reached by students is that the web seems to amplify the phenomenon of body shaming because targeting a profile on social networks involves an easier denial of individual responsibility than what would happen “offline”.

The second group chose the phenomenon of family influencers, the comments analyzed were categorized into the following types: approval, criticism, distrust, identification, empathy, familiarity, affection-belonging. In this group, students highlighted how, within these communities of family influencers, there are forms of excessive identification, followers feel part of the family, asking to be more involved and looking for attention, others create online pages dedicated to the dissemination of family influencer posts and others still use nicknames to identify them (Ferragnez, etc.).

The third group chose memes: that is, texts, images or videos combined and altered to express an opinion or to convey information, often in an ironic and satirical manner. The data collection started from some pages and groups on Facebook.

The students highlighted how the meme represents a new tool for disseminating information that takes on different characteristics. It would seem to represent the tendency of young people not to use exclusively the information channels on TV, addressing in particular the new generations and sometimes succeeding in replacing conventional sources of information. The students emphasized the symbolic value of the meme, which seems to have a dual function: on the one hand it has a playful purpose, on the other it becomes an instrument of social and political criticism.

The fourth group analyzed the practice of street art to show the different nuances of the phenomenon by describing its modalities and representations, whether concrete or abstract, representing a human condition, attempting to transmit an ideology, a point of view, a perspective or simply trying to give life to the most interstitial places of the city. The students examined some photos published on the profiles of the most followed street art artists, then grouped some of the images into 3 macro categories: fantasy and concrete images, human condition and embellishment of the city. The analysis highlighted how the mural becomes a vehicle for collective messages to raise awareness about social issues.

At the end of the task the students presented their work by building research reports and presentations that were shared in a final online meeting.

## 4. The impact of the research activity on the students involved

At the end of the exercise, students were asked to produce documents that took into account some aspects of the work carried out: this allowed to detect an array of information to understand the impact of the work done during the course.

In particular, the students had to take into account the following thematic areas: expectations toward the project work; difficulties encountered in teamwork; difficulties faced doing the ethnography in practice; learning acquired from group work; learning acquired in doing research in a digital environment; effects of observing the world of young people through the ethnographic approach; skills acquired through the realization of digital ethnography.

Let us now analyse in detail the individual areas covered in the documents.

### 4.1. Expectations

At the beginning of the didactic exercise, the students showed the expectations related to the possibility of empirically experimenting what they learned during the lectures. Being able to work within the group then created the condition to enter into a relationship with their course colleagues, testing their skills and their ability to interact with each other to reach a common goal:

*From the very beginning, I considered the research experience a means to concretize notions, concepts and perspectives treated only theoretically during the lessons. Consequently, I had high expectations that ranged from the positivity of working in a group to the application of the ethnographic method online, which has always fascinated me, and therefore, to date, I can say that those predictions have been more than satisfied (doc2)*.

*Expectations for project work were very high and were met. In my mind I started thinking about how youth practices manifested themselves in society; how do young people communicate? Is there a difference between the various generations in terms of language, and in the way of communicating? Thanks to the project work, all these questions have been clarified, especially thanks to the ethnographic approach in a digital environment (doc3)*.

*My expectations of “experimenting” have been met, because I find it fundamental to get involved in understanding when the method has actually been internalized (doc9)*.

*The difficulties encountered... in teamwork*

Despite the overall positive climate, one of the difficulties mentioned by students concerns the management of relations within the group, particularly as regards the diversity of points of view and knowledge acquired with respect to the phenomenon under consideration.

Students need to develop a positive relationship within the group and create an internal balance to facilitate the comparison of different points of view and create a collaborative action:

*I have learned that one of the difficulties of teamwork is to know how to rattle off the ideas of each member, how much they can be productive for the job and then put them into practice. A further difficulty was to reconcile the commitments of each member so that everyone could be present at the meetings and carry out the work together (doc1)*.

*Working in a group means looking at different ways of seeing the “young”, because everyone has a personal experience of how the young person relates to social networks. For this reason it can be difficult to agree on what is right or wrong (doc7)*.

*The idea of working in a group was a challenge, mainly because it was necessary to organize in a new way (online) with new people, since the course was scheduled in the first semester of the first year of distance learning. In fact, in the end this has allowed us to meet more easily and more frequently, not being bound by being in a place on a specific day, even our differences in the end have proved to be a favorable factor, since from time to time, having different experiences, contributed to enrich the work with new ideas (doc9)*.

*The difficulties encountered ... in applying the method*

Applying the method brought out one of the most widespread difficulties that a researcher faces. The activity of observing focused on a more general theme, which has always been crucial for social research, that of the relationship between researcher and social actor, of the emotional distance between the sociologist and the human context. In fact, the ethnographic research carried out by the students gave them the chance to perceive a 'forced' distance between the observed and the observer, precisely because the students, shortly before, were themselves part, as users, of that context that they now analyzed as external observers:

*Perhaps the greatest difficulty was identifying and categorizing those behaviors and attitudes that for us constitute our daily life, our lifestyles. Perhaps before now I had not associated some behaviors with keywords that best express their latent dimension and the cultural process that led to their manifestation (doc6)*.

*The main difficulty was in looking and analyzing from a scientific point of view what we observe on social networks every day; it was difficult to demonstrate that the sharing of a post by a young person is linked to cultural elements of the youth world. Another difficulty was that of managing the enormous amount of material we found online (doc7)*.

*About the application of the ethnographic method in the digital environment, a new task for us, at the beginning we felt disoriented because we did not know how to organize everything and we had doubts about the success of the final work. But thanks to the advice of the lecturers the information given to us during the lessons and the comparison with the other students in other groups, the work began to take shape. As during the lessons we showed the work done, having corrections but also positive feedback was fundamental to be able to continue (doc5)*.

*Learning acquired ... from group work*

Working in a group allowed students to experience the possibility of drawing knowledge and solutions from their peers. Each student stated that they had made themselves useful with the skills they possessed and had best contributed in the various stages leading up to the project. The debate and discussion with peers was a means by which to freely express their potential and obtain positive feedback within the group. The feeling of trust that was created was satisfactory - the combination of the different strengths developed managed to create cohesive and confident groups. In a nutshell, the learnings acquired refer to the ability to face obstacles and create joint solutions, which helped students feel able to carry out the work assigned to them. Participants also said they developed soft skills and problem solving skills that they became aware of only after finishing the job. In this sense, the ability of informal opportunities to generate learning was confirmed, as students learned “by doing together”, observing and interacting with their colleagues:

*The opportunity to share the work with other people allowed me to compare different opinions with respect to my opinions and beliefs, this allowed me to discover new perspectives and above all to be able to re-move to different questions born during work, thanks to the debate with the other members (doc1)*.

*Working in a group I personally developed a more self-critical spirit that allowed me to broaden my knowledge through different perspectives. From my point of view there were no problems both for how much look at the work within the group both with regard to the goal we had to achieve (doc4)*.

*The learning acquired working in a team was linked to the opportunity to develop and implement problem-solving techniques improving the soft skill of problem-solving (this happened in a way that was sometimes unconscious). The sharing of previous knowledge by each of individual allowed each of us to discover something more about that individual, but above all to discover that the toolbox that each of us brings with them contains different elements. This diversity has been the strong point for the emergence of new ways of observing phenomena of interest (doc8)*.

*Learnings acquired… from the applied method*

The digital ethnography method allowed students to approach a completely new methodology that gave them the possibility to understand the importance of observation and details in the analysis of social phenomena in a digital environment.

Analyzing the photos, posts and comments of members of online communities allowed them to categorize symbols, recurring themes and representations of youth cultures, managing to achieve a fairly deep understanding of certain online practices (Sassatelli, 2011; Gemini, 2015). The information that young people collected through the ethnographic method online made it possible to understand that if on the one hand, the development and evolution of social interactions is anchored to the online environment, on the other hand it can convey information and adopt cultural products that can be spread in the “offline” world (Bolander and Locher, 2020; Codeluppi, 2022; Pin, 2022):

*The challenge was to deal with a new working methodology. The ethnographic approach has allowed me to observe multiple worlds of young people who are constantly evolving, each with its own cultural frame and above all to compare them, find differences and similarities even with my world of belonging (doc1)*.

*Observing youth practices online has allowed me to understand more and more that the actions put in place by young people are unfiltered, but above all that they deserve a non-detached interpretation, a non-sententious observation look aimed at bringing out the point. of sight or perspective that hides behind what is visible (doc8)*.

*The effect of observing the world of young through the ethnographic approach*

Analyzing from a sociological point of view the youth practices that come to life online seems to have generated a positive impact on the young people involved.

Having experienced the dynamics of group work in a context of “discovery” of the ethnographic method online and techniques in digital spaces, facilitated young students to reflect on the importance of the method, as well as on their active involvement. Furthermore, in the final part of the work, the possibility for each group to present the results of the research made them the protagonists of a further comparison that led to broadening the knowledge on the variety of youth practices.

Despite the initial difficulties linked to the novelty of the approach and method of analysis, the students were able to engage in a reflexive process, which became an instrument of awareness of the generative processes of knowledge, taking the form of “self-reflection” in which young people focus on themselves and their selves, and the practices of which they are integral part.

*Taking the role of ethnographer of the digital spaces, as a young student, has made me aware of the different strategies that can be adopted in this research context and, moreover, assured me that behind that interface that appears on the screen, there may be someone who has a specific interest in our hobbies, our preferences, our practices (doc2)*.

*Observing the worlds of young people through the ethnographic approach and immersing ourselves in their lives was at first curious and unusual, as we had never observed and analyzed their lives in such an assiduous and in-depth way. Before we were asked to do this job, we followed family influencers but rarely stopped to reflect on their photos and related comments (doc5)*.

*I managed to develop a more critical attitude, paying attention to what I didn't give much importance to before. This led me to an inner and professional growth. I wore some new lenses with which to observe some phenomena (doc6)*.

### 4.2. Acquired skills

Among the skills that young people claimed to have acquired, are interpersonal skills, resulting from the need to arrive at conclusive reflections on the work done. Putting different points of view together through comparison and group discussion allowed students to understand that working in a group means having to interact constructively with colleagues, without trying to make one or the other point of view prevail.

Students declared that they have acquired organizational skills both in relation to the division of the work to be carried out and to put in order all the material collected. This also allowed the students to understand that ethnographic research requires methodological rigor. Studying, observing the reality in which one is inserted can be for many pay an advantage. It represents an opportunity that can add much to the cognitive process without detracting from the validity and methodological rigor of observation; it can be a situation that facilitates the work of a young novice researcher who can start doing research by trying to analyze environments in which feels safer, in which he moves more easily, and then “distracts” by entering situations that are more alien to him (Quarta, 2020):

*Thanks to the realization of the project through digital ethnography we were able to acquire different skills: first of all we learned to set up an ethnographic research, respecting all its phases; we learned the importance of details; it was important to observe the place and time when the photo was taken. All these details were new to us because we did not know they could be decisive for the purpose of the analysis (doc5)*.

*Through the realization of the project work I think I have acquired greater critical thinking skills and familiarity with the tools suitable for an accurate analysis of digital platforms (doc6)*.

A reflection that is worth making after analyzing the students' reports is that their testimonies show a strong appreciation for having empirically carried out the research work, thus experiencing the knowledge learned during the lectures. Having done so in a field of investigation that represents an important sphere of their daily life has meant learning to see the social and cultural reality to which they belong from a different, perhaps even more detached, point of view, which certainly led to reflect on their lifestyles and the daily practices in which they are involved.

## 5. Conclusions

New digital technologies constitute a crucial node in the social dynamics of young people, they mark the rhythms of their daily lives, they invade much of the communicative, cognitive and creative experiences they carry out (Marwick and Boyd, 2014).

The results of the laboratory experience carried out by the students of the University of Salerno confirm this inclination, showing how much the rooting and pervasiveness of digital media involves dynamics and practices that underlie the relationships and experiences of young people, who grow up and interact in a social and cultural fabric that places the media at the center of most of the experiences that concern their sociality (Zaalberg et al., 2004; Riva and Scarcelli, 2016; Turkle, 2019). Young people who are part of the same community (such as family influencers or street artists) or who are able to interpret and decode the same communicative symbols (as in the case of memes) share values, interests, passions, exchange interactions that are not opposed to those formed in face-to-face encounters in everyday life but integrate with them (Rheingold, 2000; Preece and Maloney-Krichmar, 2003; Anderson, 2004; Drusian et al., 2019), through dynamics that confuse and overlap online and offline contexts. These are relational spaces mediated or not mediated by technologies, which define new scenarios of interaction which might seem less dense and binding than traditional ones but which nonetheless provide a way to obtain social recognition and identification.

The results show, first of all, how the method applied allows to analyze the contours and transformations of youth practices in digital spaces, and secondly it permits to instigate “reflexivity” in young people: students looked at themselves "in the mirror”, entering the folds of the virtual worlds of young people and were amazed at the way they represent themselves, becoming observers of that same reality.

As for the impact of ethnographic work on young sociologists, it was found to be overall positive, both in terms of expectations and acquired learning. The students had the opportunity to use the digital ethnography method for the first time, facing difficulties related to both the management of relationships within the group and the experimentation of the method used during the research.

There was no lack of difficulties related to the management and organization of time and work tasks, in a context that, although overall favorable, envisaged the need to articulate and reshape the initial projects, undergoing progressive corrections and adjustments. In addition to this is the fact of having to apply the ethnographic method online to the study of youth practices very close to them. From this point of view, the difficulty was twofold: on the one hand, getting involved in experimenting with a social research method; on the other, having to distance themselves from practices that mark their daily lives, in order to be able to study their contours and peculiarities in a neutral way.

Working in a group led the students to reflect on their agency and problem-solving skills; having experimented with the ethnographic method online allowed them to deal with an approach that they themselves define as interesting and that they believe they want to use for future research as well.

Via this experience, the students were able to grasp the youth practices of which they are part in their daily life from another point of view (Gardner and Davis, 2014; Furin and Longo, 2019); they had the opportunity to analyze them from the inside and develop a high degree of reflexivity that helped them to reach a different awareness of what being young today and being able to critically read the cultural dynamics of youth, actually means.

## Data availability statement

The original contributions presented in the study are included in the article/supplementary material, further inquiries can be directed to the corresponding author.

## Ethics statement

Ethical approval was not required for the study involving human participants in accordance with the local legislation and institutional requirements. Written informed consent to participate in this study was provided by the participants.

## Author contributions

The author confirms being the sole contributor of this work and has approved it for publication.

## References

[B1] Altheide D. L. (2000). L'analisi Qualitativa dei Media. Soveria Mannelli: Rubbettino.

[B2] Amendola A. Castellano S. Troianiello N. (2020). #likeforlike. Categorie, Strumenti e Consumi Nella Social Media Society. Roma: Rogas.

[B3] Anderson B. (2004). Dimensions of learning and support in an online community. Open Learn. J. Open Dist. e-Learn. 19, 183–190. 10.1080/0268051042000224770

[B4] Anne-Marie T. Chau N. Kai K. K. (2017). Ethical questions related to using netnography as research method. The ORBIT J. 1, 1–11. 10.29297/orbit.v1i2.5035657652

[B5] Bakardjieva M. (2005). Internet Society: The Internet in Everyday Life. London: Sage.

[B6] Beneiro-Montagut R. (2011). Ethnography goes online: towards a user centred methodology to research interpersonal communication on the internet. Qual. Res. 11, 716–735. 10.1177/1468794111413368

[B7] Boccia Artieri G. (2011). Forme e pratiche della socievolezza in Rete: connessi in pubblico. Sociol. Della Comun. 42, 51–66. 10.3280/SC2011-041005

[B8] Bolander B. Locher M. A. (2020). Beyond the online offline distinction: entry points to digital discourse. Discours. Cont. Media 35, 100383. 10.1016/j.dcm.2020.100383

[B9] Chambers D. (2013). Social Media and Personal Relationships: Online Intimacies and Networked Friendship. Basingstoke: Palgrave Macmillan.

[B10] Chiriatti M. (2021). L'osservazione partecipante come tecnica di ricerca sociale: dal metodo tradizionale al web 2, 0 (Bachelor's thesis). University Luiss Guido Carli, Rome, Italy.

[B11] Codeluppi V. (2022). Mondo Digitale. Rome: Gius, Laterza and Figli Spa.

[B12] Costello L. McDermott M. L. Wallace R. (2017). Netnography: range of practices, misperceptions, and missed opportunities. Int. J. Q. Methods 16, 1609406917700647. 10.1177/1609406917700647

[B13] Drusian M. Magaudda P. Scarcelli M. (2019). Vite interconnesse. Pratiche digitali Attraverso app, Smartphone e Piattaforme Online. Milano: Meltemi.

[B14] Esposito M. (2016). La netnografia. Strumenti e tecniche per la ricerca etnografica online (Doctoral thesis). University of Salerno, Fisciano, Italy.

[B15] Furin A. Longo M. (2019). Esplorando il sottile confine tra reale e virtuale. Gruppi 1, 17–29. 10.3280/GRU2019-001003

[B16] Gardner H. Davis K. (2014). Generazione App. La Testa dei Giovani e il Nuovo Mondo Digitale. Milano: Feltrinelli.

[B17] Gemini L. (2015). Visual Networking. Appunti Sulla Dimensione Visuale dei Media Sociali, Gli Effetti Sociali del Web. Forme Della Comunicazione e Metodologie Della Ricerca Online. Milano: FrancoAngeli, 105–122.

[B18] Hine C. (2000). Virtual Ethnography. London: Sage.

[B19] Kozinets R. V. (2002). The field behind the screen: using netnography for marketing research in online communities. J. Market. Res. 39, 61–72. 10.1509/jmkr.39.1.61.18935

[B20] Kozinets R. V. (2006). Netnography. Handb. Q. Res. Methods Market. 129, 142. 10.4337/9781847204127.00018

[B21] Kozinets R. V. (2019). Netnography: The Essential Guide to Qualitative Social Media Research. London: Sage.

[B22] Lupton D. (2018). Sociologia Digitale. Torino: Pearson.

[B23] Marwick A. E. Boyd D. (2014). Networked privacy: how teenagers negotiate con-text in social media. New Media Soc. 16, 1051–1067. 10.1177/1461444814543995

[B24] Masullo G. Addeo F. Delli Paoli A. (2020). Etnografia e Netnografia. Ri- Flessioni Teoriche, Sfide Metodologiche ed Esperienze di Ricerca. Napoli: Loffredo.

[B25] Pattaro C. Setiffi F. (2020). La Socialità Mediata: Strategie e Modalità Comunicative Degli Adolescenti tra Online e Offline. Milano: Vita e Pensiero.

[B26] Pin A. (2022). Nella Rete, anche se offline. Il ruolo dello spazio pubblico nell'era digitale. Mondo Digit. 98, 1–9.

[B27] Preece J. Maloney-Krichmar D. (2003). Online communities: focusing on sociability and usability. Handb. Hum. Comput. Int. 2, 596–620.

[B28] Quarta S. (2020). L'osservazione Partecipante: Uno Strumento di Conoscenza Della Complessità Sociale. Milano: Ledizioni.

[B29] Rheingold H. (2000). The Virtual Community, Revised Edition: Homesteading on the Electronic Frontier. London: MIT Press.

[B30] Riva C. Scarcelli C. M. (2016). Giovani e Media. Milano: McGraw-Hill.

[B31] Romeo A. (2017). Posto, Taggo, Dunque Sono? Nuovi Rituali e Apparenze Digitali. Sesto San Giovanni: Mimesis.

[B32] Ruhleder K. (2000). The virtual ethnographer: fieldwork in distributed electronic environments. Field Methods 12, 3–17. 10.1177/1525822X0001200101

[B33] Sassatelli R. (2011). Cultura visiva, studi visuali. Stu. Cult. 8, 147–154.

[B34] Savonardo L. (2020). GenerAzioni Digitali: Teorie, Pratiche e Ricerche Sull'universo Giovanile. Milano: Egea.

[B35] Tirocchi S. (2013). Sociologie della Media Education. Giovani e Media al Tempo dei Nativi Digitali. Milano: FrancoAngeli.

[B36] Turkle S. (2019). Insieme ma Soli. Perché ci Aspettiamo Sempre piú Dalla Tecnologia e Sempre Meno Dagli Altri. Torino: Einaudi.

[B37] Zaalberg R. Manstead A. S. R. Fischer A. H. (2004). Relations between emotions, display rules, social motives, and facial behaviour. Cognit. Emot. 18, 183–207. 10.1080/02699930341000040 29148304

